# The *Chlamydia trachomatis* effector CT226 directly engages LRRFIP1 to recruit a multiprotein complex that limits inclusion size and progeny production

**DOI:** 10.1128/mbio.01953-26

**Published:** 2026-09-17

**Authors:** Cherilyn A. Elwell, Amy Diallo, Barbara S. Sixt, Jessica Sherry, Khavong Pha, Raphael H. Valdivia, Oren Rosenberg, Kliment Verba, Joanne Engel

**Affiliations:** 1 University of California San Francisco 8785, San Francisco, California, USA; 2 Duke University Medical Center 609772, Durham, North Carolina, USA; NYU Langone Health, New York, New York, USA

**Keywords:** *Chlamydia trachomatis*, intracellular bacteria, host–pathogen interactions, vacuolar bacteria, inclusion membrane protein, LRRFIP1, LRRFIP2, Flightless, TMOD, microbial cell biology, alphafold

## Abstract

*Chlamydia* species secrete a unique class of effector proteins termed inclusion membrane proteins (Incs) that are translocated into the inclusion membrane and reprogram host cell processes to support intracellular growth. Using affinity purification–mass spectrometry, we previously identified an interaction between the early expressed Inc CT226 and a human multiprotein complex comprising leucine-rich repeat Flightless-I-interacting protein 1 (LRRFIP1), LRRFIP2, Flightless-I (FLII), and tropomodulin 3 (TMOD3). Here, we define the molecular basis and functional consequences of this interaction during *Chlamydia trachomatis* infection. We show that CT226 is both necessary and sufficient to recruit the LRRFIP1/2:FLII:TMOD3 (LFT) complex to the inclusion membrane. *In vitro* reconstitution with purified proteins demonstrates that the predicted coiled-coil region of CT226 directly binds the N-terminal helix of LRRFIP1. Using *in silico* structural modeling with AlphaFold-Multimer coupled with mutational validation, we identify key residues that mediate an electrostatic CT226:LRRFIP1 interaction required for LFT recruitment to the inclusion. Unexpectedly, genetic disruption of CT226 or its interaction with the LFT complex results in modest increases in inclusion size and recovery of infectious progeny late in infection, demonstrating that the CT226:LFT interaction modulates *C. trachomatis* developmental progression. Together, these findings establish the molecular basis of CT226-mediated recruitment of the LFT complex to the inclusion membrane and uncover a previously unrecognized role for this effector–host interaction at late timepoints of the *C. trachomatis* intracellular developmental cycle.

**IMPORTANCE:**

*Chlamydia trachomatis* is a major cause of sexually transmitted infections and blindness worldwide. This bacterium survives by replicating inside host cells within a membrane-bound compartment, where it deploys specialized proteins to manipulate host processes. These proteins, known as inclusion membrane proteins (Incs), help organize the host–pathogen interface, but the molecular mechanisms by which many Incs engage host factors remain poorly understood. Here, we show that the Inc protein CT226 recruits a host multiprotein complex to the bacterial compartment by directly binding one of its components. Using structural modeling and targeted mutations, we define the molecular interface required for this interaction. Disruption of CT226 or its ability to engage this host complex results in modest increases in inclusion size and production of infectious progeny, suggesting a role for the CT226:host interaction in regulating *Chlamydia* intracellular development. These findings reveal how a bacterial effector can engage a host protein complex to influence the outcome of infection.

## INTRODUCTION

*Chlamydia* species are obligate intracellular pathogens that are important causes of human disease (1). *Chlamydia trachomatis* serovars cause ocular and genital infections that, if left untreated, can lead to chronic inflammation and devastating sequelae, including blindness and sterility (1). In 2023, *C. trachomatis* remained as the most commonly reported sexually transmitted bacterial infection in the United States, with over 1.6 million reported cases and the highest burden among adolescents and young adults (2). A deeper understanding of the *C. trachomatis* pathogenesis is essential for the development of a successful vaccine, which to date remains unavailable.

All *Chlamydia* species share a common intracellular developmental cycle in which they alternate between an infectious elementary body (EB) and a non-infectious, replicative reticulate body (RB) (3). Following binding, EBs are internalized via receptor-mediated endocytosis into a membrane-bound compartment—the inclusion—which rapidly diverges from the canonical endo-lysosomal pathway. Within the inclusion, EBs differentiate into RBs and initiate replication. Over the next 24–72 h, RBs proliferate within the expanding inclusion before undergoing secondary differentiation back into EBs. Mature EBs are then released either through host cell lysis or by an active extrusion process, enabling infection of neighboring cells.

As part of its intracellular survival strategy, *Chlamydia* encodes a type III secretion system (T3SS) that delivers two major classes of bacterial effectors (4, 5): (i) soluble effectors that enter the host cytosol with access to various host intracellular compartments; and (ii) inclusion membrane proteins (Incs), a specialized family of membrane proteins unique to the phylum Chlamydiota, that are inserted post-translationally into the inclusion membrane during infection. A defining feature of the Incs is the presence of one or more bilobed hydrophobic regions composed of two closely spaced membrane-spanning domains typically separated by a short loop region in the inclusion lumen (6, 7). Once inserted into the inclusion membrane, both the N- and C-termini of Incs are predicted to extend into the host cytoplasm (6), strategically positioning them at the host–pathogen interface to mediate interactions between the inclusion and host cell machinery. These host–pathogen interactions license the pathogen to modulate key host processes, including cytoskeletal dynamics, vesicle trafficking, gene and protein expression, nutrient acquisition, the cell cycle, and immune evasion.

Advances in high-throughput proteomics of host–pathogen interactions (8–10) together with recent progress in *C. trachomatis* genetics (11) have enabled systematic identification and functional analysis of Inc-host interactions during *C. trachomatis* infection. Using high-throughput affinity purification–mass spectrometry (AP-MS), we previously generated a global *C. trachomatis* serovar D Inc-human interactome. This screen identified a predicted high-confidence interaction between the *C. trachomatis* Inc CT226 and a multiprotein complex comprising leucine-rich repeat Flightless-I-interacting protein 1 (LRRFIP1), its paralog LRRFIP2, Flightless-I (FLII), and tropomodulin 3 (TMOD3) (hereafter referred to as the LFT proteins or the LFT complex for LRRFIP1/2:FLII:TMOD3) (12). CT226 is an early expressed Inc (13) of unknown function consisting of a short cytosolically exposed N-terminus with the type III secretion signal, two predicted transmembrane domains, and a cytosolically exposed C-terminus. Although CT226 shares no detectable homology with known bacterial or eukaryotic proteins, structural predictions indicate that its C-terminal region contains a coil–coil (CC) domain (Fig. S1A), suggesting a potential structural interface for host complex binding that we set out to experimentally define and functionally characterize.

The LFT proteins are multifunctional host proteins that have been implicated in integrating cytoskeletal regulation, signaling, and innate immune responses (14–16), making them attractive targets for microbial manipulation. LRRFIP1 and its paralog LRRFIP2 were initially characterized through their interaction with FLII (17). LRRFIP1 has been reported to contribute to the Wnt/β-catenin pathway, TLR-MyD88-NF-κB signaling, transcriptional regulation, and innate immune defense, where it serves as an adaptor in nucleic acid sensing pathways to promote type I interferon production (14). LRRFIP2 not only shares overlapping functions (14) but has also been implicated in the regulation of the inflammasome activity (15). FLII, a member of the gelsolin family, not only possesses actin-remodeling activity but also functions as a transcriptional co-regulator in development, cell migration, wound repair, inflammation, and immune responses (15). TMOD3, an actin-capping protein, regulates filament length and stability, thereby contributing to vesicular trafficking and cellular organization (16). Together, these proteins form a higher-order complex that potentially integrates cytoskeletal dynamics with transcriptional and innate immune responses (14–16), highlighting their roles as critical host factors at the pathogen–host interface.

In this study, we combine genetics, biochemistry, structural modeling, and microscopy to delineate the molecular basis and functional consequences of the CT226:LFT interaction during infection. We constructed a CT226 insertion mutant and demonstrate that this Inc is necessary to recruit the LFT complex to the inclusion. We further show that the predicted CC of CT226 is both necessary and sufficient to bind directly to the N-terminal helix of LRRFIP1. We used structure-guided mutagenesis to identify key interface residues whose disruption abolishes the CT226:LRRFIP1 interaction and prevents recruitment of the LFT complex to the inclusion membrane. Finally, genetic disruption of CT226 or its interaction with the LFT complex leads to modest increases in inclusion size and recovery of infectious progeny at late stages of infection. Together, these findings support a model in which the CT226 CC domain captures the N-terminal helix of LRRFIP1, and likely LRRFIP2, through electrostatic interactions, thereby nucleating assembly and relocalization of the LFT complex to the inclusion membrane. These protein–protein interactions at the host–pathogen interface modulate the intracellular developmental cycle at late time points.

## RESULTS

### CT226 interacts with LFT proteins during infection

Our AP-MS screen identified a high-confidence interaction between CT226 from *C. trachomatis* serovar D expressed in HEK293T cells and the human multiprotein LFT complex (12). Because genetic tools are most developed for *C. trachomatis* serovar L2 (strain LGV434/Bu; hereafter referred to as L2) (11, 18), and because CT226 is highly conserved between serovars D and L2 (98% identity at the amino acid sequence; homolog of CT226 in L2 is CTL0478), we conducted all subsequent studies with L2.

**Fig 1:**
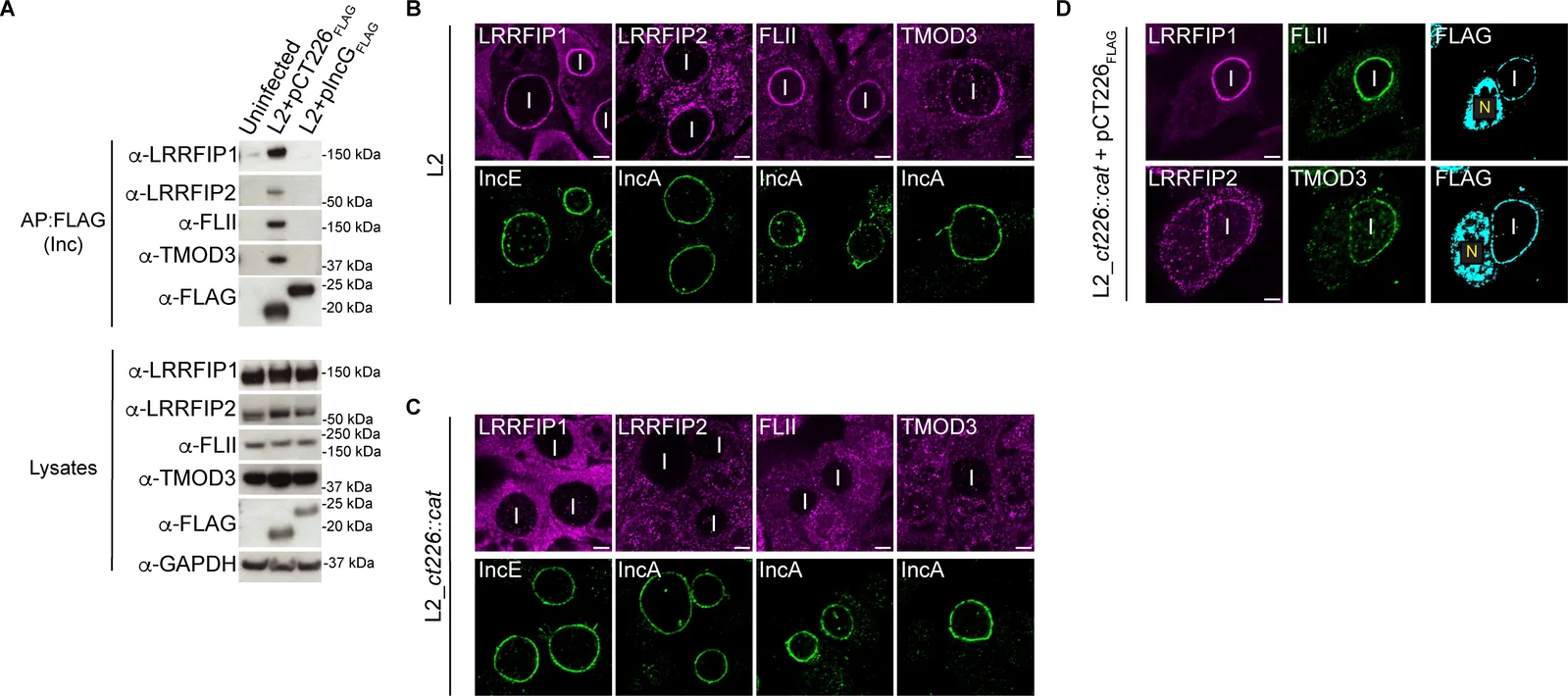
CT226 interacts with the LFT complex and is required for its recruitment to the inclusion membrane. (**A**) HeLa cells were infected with either L2 expressing CT226_FLAG_ or IncG_FLAG_ for 24 h. Protein expression was induced at 1 hpi with aTc. FLAG-tagged Incs were affinity purified, and both lysates and eluates were analyzed by immunoblotting with antibodies to endogenous LFT proteins or to FLAG (Inc). GAPDH serves as a loading control. Molecular weight markers (kDa) are indicated on the right. The LFT proteins co-purified with CT226 but not with IncG, indicating a specific interaction. (**B through D**) HeLa cells were infected with either (**B**) L2, (**C**) L2_*ct226::cat*, or (**D**) L2_*ct226::cat* + pCT226_FLAG_. Protein expression was induced at 1 hpi with aTc. Cells were fixed at 24 hpi and stained with antibodies to (**B and C**) endogenous LFT proteins (magenta) and IncE or IncA (green) to delineate the inclusion membrane. (**D**) Cells were stained with antibodies to LRRFIP1 or LRRFIP2 (magenta), FLII or TMOD3 (green), and FLAG for CT226_FLAG_ (cyan). Non-specific nuclear staining is observed with the FLAG antibody. Samples were imaged by spinning disk confocal microscopy. Shown are single Z-slices. I, inclusion; N, nucleus. Scale bar = 5 µm.

To confirm that CT226 interacts with the LFT proteins during *C. trachomatis* infection, we performed co-affinity purification studies in HeLa cells infected with L2 transformed with a plasmid expressing CT226 fused to a FLAG tag (L2 + pCT226_FLAG_). As a specificity control, HeLa cells were infected with an L2 strain expressing FLAG-tagged IncG (L2 + IncG_FLAG_), an unrelated Inc. Endogenous LFT proteins co-affinity purified with CT226_FLAG_ but not with IncG_FLAG_ (Fig. 1A). These findings confirm that CT226 interacts specifically with the LFT complex during infection.

### The LFT proteins are recruited to the inclusion in a CT226-dependent manner

To determine whether CT226 directly recruits the LFT proteins to the *C. trachomatis* inclusion, we generated a CT226 insertion mutant using the TargeTron technology (19). A chloramphenicol acetyltransferase cassette (CAT) was inserted at amino acid 138 within the predicted CC domain (Fig. S2A), which was validated by PCR (Fig. S2B). To confirm whether the observed phenotypes were attributable to loss of CT226, rather than possible polar effects on neighboring genes, we complemented L2_*ct226::cat* by transforming it with pCT226_FLAG_, generating L2_*ct226::cat*+pCT226_FLAG_.

Using these isogenic strains, we tested whether CT226 is necessary and sufficient for recruiting the LFT proteins to the inclusion. Immunofluorescence microscopy showed robust recruitment of the endogenous LFT proteins to inclusions in L2-infected cells but not to inclusions in cells infected with L2_*ct226::cat* (Fig. 1B and C). Infection with the complemented mutant (L2_*ct226::cat*+p226_FLAG_) restored recruitment of LFT proteins to the inclusions (Fig. 1D), demonstrating that CT226 is both necessary and sufficient for recruitment of the LFT proteins to the inclusion.

### The predicted CC domain in CT226 is necessary and sufficient for binding to the LFT proteins

**Fig 2:**
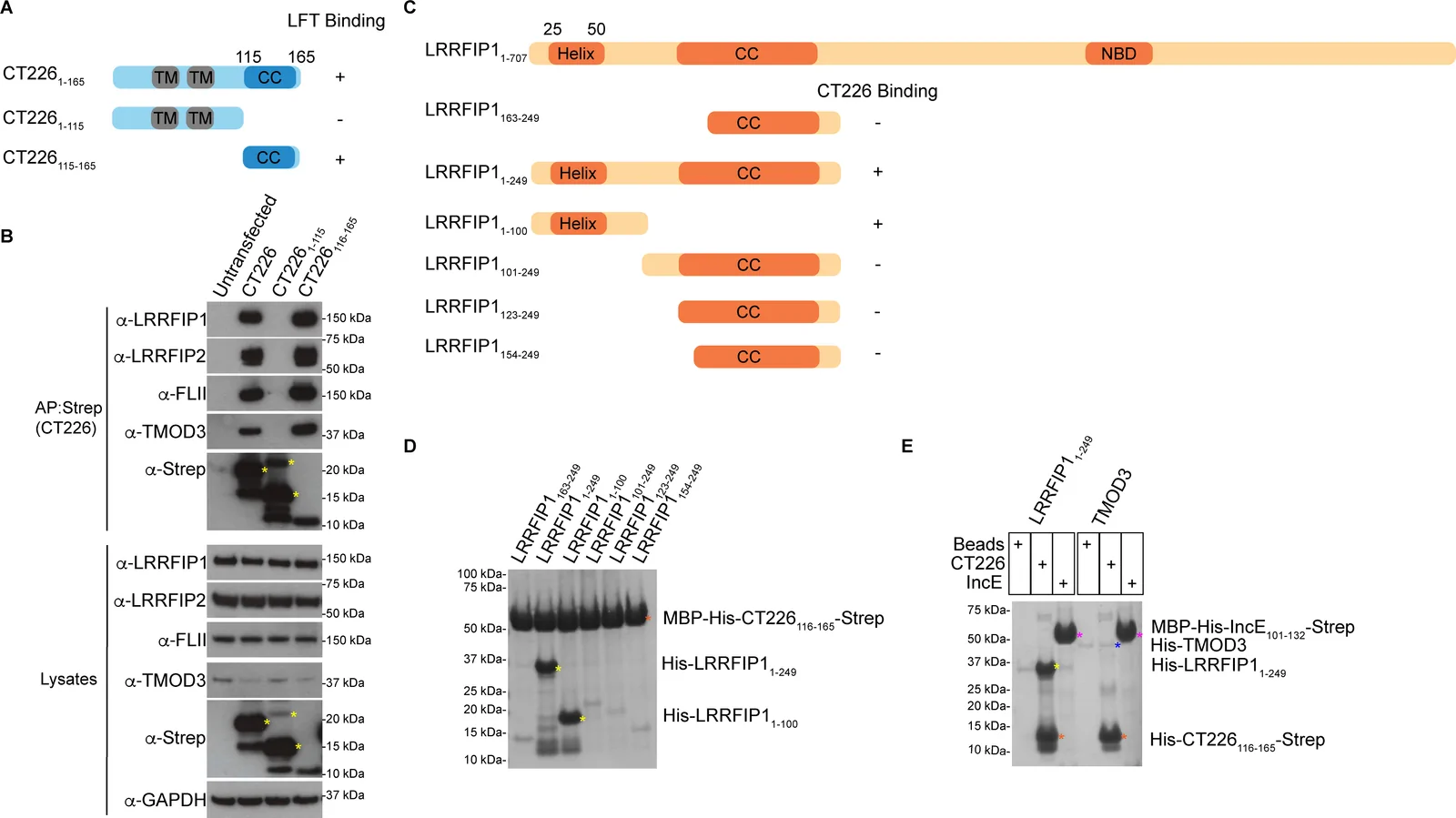
The CT226 CC domain interacts directly with the LRRFIP1 N-terminal helix. (**A**) Schematic representation of CT226 constructs annotated with corresponding amino acid residues and domain structures. TM, transmembrane; CC, coiled coil. A summary of binding results is shown alongside the schematic. (**B**) Affinity purification of Strep-tagged CT226 truncation variants transiently expressed in HEK293T cells, followed by immunoblotting with the indicated antibodies. CT226 multimers are indicated with yellow asterisks. GAPDH serves as a loading control for the lysates. The CT226 CC domain was necessary and sufficient for interacting with the LFT proteins. Molecular weight markers (kDa) are indicated on the right. (**C**) Schematic representation of LRRFIP1 constructs annotated with corresponding amino acid residues and domain structures. CC, coiled coil; NBD, nucleotide-binding domain. A summary of binding results is shown alongside the schematic. (**D**) Purified Strep-tagged CT226_116-165_ fused to maltose-binding protein (MBP) (orange asterisk) was immobilized to Strep-Tactin beads and incubated with purified His-tagged LRRFIP1 truncation variants (yellow asterisks). Eluates were resolved by SDS-PAGE and visualized by Coomassie blue stain. MBP-His-CT226_116-165_-Strep (orange asterisk) binds directly and specifically to His-LRRFIP1 constructs containing the N-terminal helix (yellow asterisks). Molecular weight markers (kDa) are indicated on the left. Input shown in Fig. S3A. (**E**) Purified Strep-tagged CT226_116-165_ fused to His (orange asterisk) or purified Strep-tagged IncE_101-132_ fused to MBP (pink asterisk) was immobilized to Strep-Tactin beads and incubated with His-tagged LRRFIP1_1-249_ (yellow asterisk) or His-tagged TMOD3 (a blue asterisk indicates faint background band of TMOD3 binding to beads and CT226). Complexes were resolved by SDS-PAGE and visualized by Coomassie blue stain. His-CT226_116-165_-Strep (orange asterisk) binds directly and specifically to His-LRRFIP1_1-249_ (yellow asterisk). No interaction above beads alone was detected between His-CT226_116-165_-Strep (orange asterisk) and His-TMOD3 (blue asterisk). No interaction was detected between MBP-His-IncE_101-132_-Strep (pink asterisk) and His-LRRFIP1_1-249_ (yellow asterisk). Molecular weight markers (kDa) are indicated on the left. Input shown in Fig. S3B.

Having established that CT226 interacts specifically with the LFT proteins during infection, we next sought to define the region of CT226 that is responsible for this interaction. We transfected HEK293T with various truncation mutants of CT226 fused to Strep (Fig. 2A), followed by affinity purification and immunoblotting. Endogenous LFT proteins copurified only with CT226 constructs containing amino acid residues 116–165, a segment encompassing the predicted CC domain (Fig. 2B). These results indicate that the CC domain is both necessary and sufficient for binding this multiprotein complex.

### The N-terminal 100 amino acids of LRRFIP1 are necessary and sufficient to bind directly to the CT226-predicted CC domain

Next, we asked which of the LFT protein(s) bind directly to CT226_116-165_ and sought to further define their binding domains. LRRFIP1 and LRRFIP2 contain two highly conserved regions (14): (i) a short N-terminal helix of unknown function; and (ii) a central 87 amino acid CC domain required for interaction with FLII (17). In LRRFIP1, this central CC domain also mediates homodimerization (20). The proteins differ at the C-terminus: LRRFIP1 encodes a predicted nucleic acid-binding domain, whereas LRRFIP2 contains an additional CC domain. FLII comprises two major domains (15): (i) an N-terminal leucine-rich repeat region (LRR), which mediates its interaction with LRRFIP1 and LRRFIP2; and (ii) a C-terminal region with six gelsolin-like repeats. TMOD3 contains an unstructured N-terminal domain with one actin binding site and two tropomyosin-binding sites for high affinity capping of tropomyosin-coated actin filaments and a C-terminal globular domain with five leucine-rich repeats, a second actin binding site, and alpha-helix, and a C-terminal tail that mediate core pointed-end capping (16).

For these studies, we expressed and purified various versions of the LFT proteins in *Escherichia coli*. We focused on LRRFIP1 and TMOD3 because of solubility issues with LRRFIP2 and FLII proteins. Given the conservation of both the N-terminal helix and the central CC domain of LRRFIP1 and LRRFIP2, we predicted that CT226 likely interacts within LRRFIP1 residues 1–249 and, therefore, analyzed versions of LRRFIP1 constructs that lacked one or the other of these domains (Fig. 2C). CT226_116-165_ bound directly and specifically to the LRRFIP1 variants that encoded the N-terminal helix but failed to bind to variants that lacked the N-terminal helix (Fig. 2C and D; Fig. S3A). Notably, CT226_116-165_ did not bind to purified TMOD3 (Fig. 2E; Fig. S3B), suggesting that the presence of TMOD3 in the CT226:LFT complex results from TMOD3 interaction with LRRFIP1, LRRFIP2, and/or FLII as opposed to TMOD3 binding directly to CT226. Together, these results demonstrate that the CC domain of CT226 (residues 116-165) directly engages with the N-terminal helix of LRRFIP (residues 1-100).

**Fig 3:**
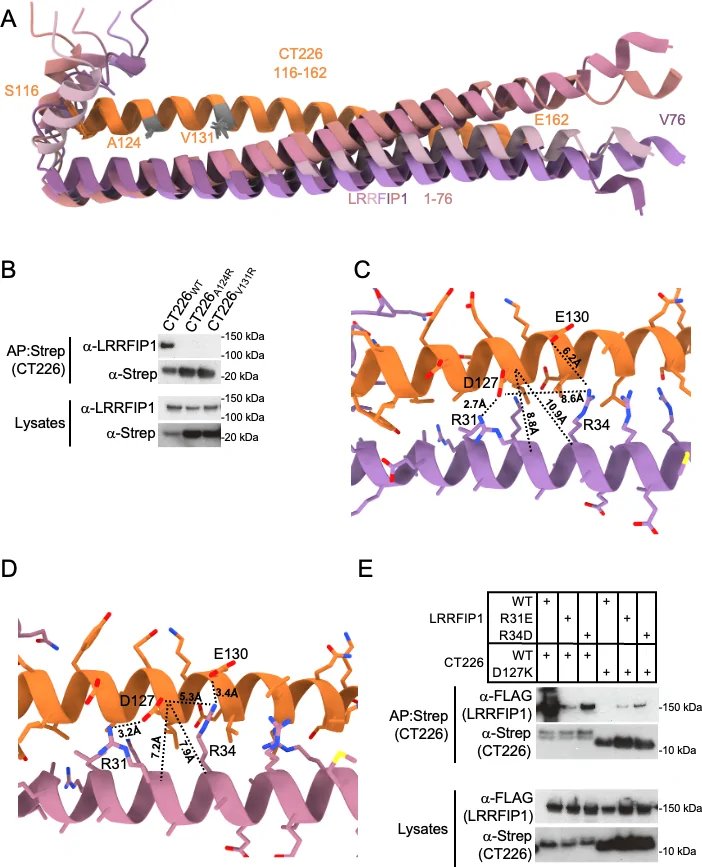
Structural modeling and experimental validation of the CT226:LRRFIP1 interaction interface. (**A**) Five models of the CT226_116-162_:LRRFIP1_1-76_ interface predicted by AlphaFold2 aligned on CT226 are shown as ribbons that are truncated at the helices to remove lower-confidence random coil regions. CT226 ribbons are in orange, and LRRFIP1 ribbons are in different shades of purple. CT226 residues A124 and V131 are shown as gray sticks. (**B**) Affinity-purified Strep-tagged CT226 single-point mutants A124R and V131R, transiently expressed in HEK293T cells, were analyzed by immunoblotting with the indicated antibodies.. Molecular weight markers (kDa) are indicated on the right. Mutation of CT226 residues A124 or V131 disrupts binding to LRRFIP1. Immunoblots of LRRFIP2, FLI-I, and TMOD3 are shown in Fig. S5A. (**C**) One of two remaining models showing a salt bridge network centered on LRRFIP1 R31 and R34. The distances between the C-alpha carbons and putative charged atoms are shown for R31-D127 and R34-D127. In this model, R31 makes a salt bridge with D127 but not with R34, which is too distant. (**D**) Last remaining model showing salt bridge network centered on LRRFIP1 R31 and R34. In this model, both R31 and R34 are close enough to D127 to form salt bridges. (**E**) Affinity purification of FLAG-tagged LRRFIP1 variants transiently co-expressed with Strep-tagged CT226 variants, followed by immunoblotting with antibodies to FLAG (LRRFIP1 variants) and Strep (CT226). Variants include charge mutations that are predicted to disrupt binding as well as charge-reversal mutations that are predicted to rescue binding based on the structures in Fig. 3C and D. Molecular weight markers (kDa) are indicated on the right. Individual charge mutations in CT226 and LRRFIP1 disrupt the CT226:LRRFIP1 interaction, while charge-reversal mutations partially restore binding of CT226._D127K_ to LRRFIP1_R31E_ and LRRFIP1_R34E_, supporting the model shown in D. For quantitation, see Fig. S5B and C.

### Structural modeling identifies residues in CT226 and LRRFIP1 that are necessary for the CT226:LRRFIP1 interaction

To further investigate the CT226:LRRFIP1 binding interface, we performed structural modeling of CT226_116-165_ and LRRFIP_1-100_ using AlphaFold-Multimer (21, 22). The top five scoring models were highly similar, each predicting a coiled-coil interaction between CT226 and LRRFIP1, indicating convergence in the predictions (Fig. 3A; Fig. S4). However, these models differed in both the angle between the alpha helices and their register. To distinguish between these models, we designed mutants targeting informative residues of LRRFIP1 and CT226 and tested their binding by transfection, followed by affinity purification and immunoblotting.

**Fig 4:**
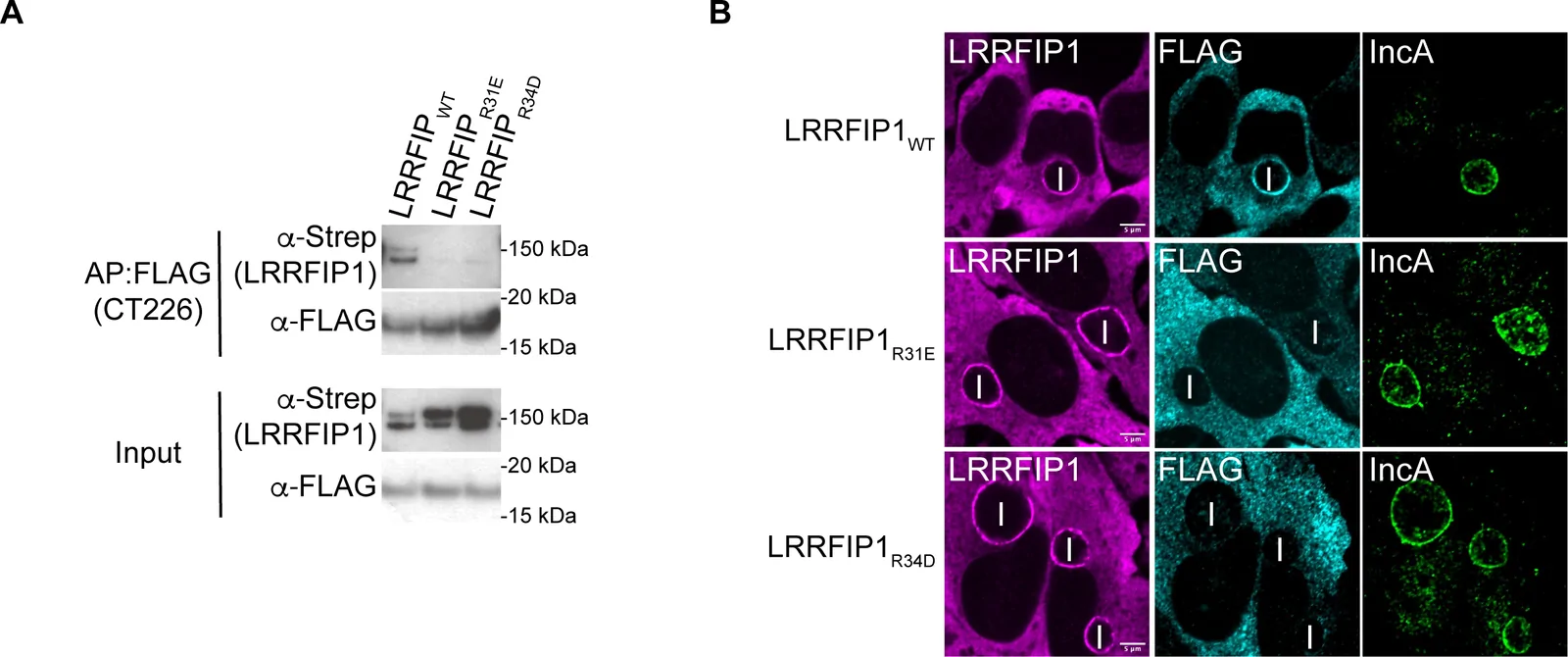
LRRFIP1 residues R31 and R34 are critical for its binding to CT226 and for its recruitment to the inclusion during infection. (**A**) HeLa cells were transfected with Strep-tagged LRRFIP1 constructs (WT, R31E, R34D), and then infected with L2 expressing FLAG-tagged CT226 for 24 h. Protein expression was induced at 1 hpi with aTc. FLAG-tagged CT226 was affinity purified, followed by immunoblotting with antibodies to Strep (LRRFIP1) and FLAG (CT226). Molecular weight markers (kDa) are indicated on the right. LRRFIP1 variants R31E or R34D disrupt binding to CT226 during infection. For all constructs, LRRFIP1 migrates as a doublet. (**B**) HeLa cells were transfected with FLAG-tagged LRRFIP1 constructs (WT, R31E, R34D), and then infected with L2 for 24 h. Cells were fixed and stained with an antibody to LRRFIP1 (magenta, which stains endogenous and FLAG-tagged LRRFIP1), FLAG (cyan, which only stains FLAG-tagged LRRFIP1), and IncA (green, to delineate the inclusion membrane). Samples were imaged by spinning disk confocal microscopy. Shown are single Z slices. Scale bar = 5 µm. Transfected FLAG-tagged LRRFIP1 but not LRRFIP1_R31E_ or LRRFIP1_R34D_ (cyan) was recruited to the inclusion membrane. Endogenous LRRFIP1_WT_ (magenta) was recruited to the inclusion membrane, regardless of the transfected FLAG-tagged LRRFIP1 variant.

Initially, we validated the overall arrangement of these models by mutating CT226 residues A124 and V131, which are predicted to pack tightly against LRRFIP1 in all models. We hypothesized that substituting these residues with arginine, which is bulky and charged, would disrupt binding due to steric hindrance (Fig. 3A). Indeed, mutation of CT226 A124 and V131 to arginine (CT226_A124R_ and CT226_v131R_) abrogated CT226 binding to LRRFIP1 (Fig. 3B), supporting the predicted interface. Mutation of these residues also reduced binding to LRRFIP2, FLII, and TMOD3 (Fig. S5A).

Next, we generated mutations in LRRFIP1 that would allow us to narrow down to a specific model(s). In three of the five models, LRRFIP1 residues L15, A23, and A37 are predicted to pack tightly against CT226 and are expected to be intolerant to substitution with larger residues (Fig. S6A). However, replacing each of these residues with glutamic acid (LRRFIP1_L15E_, LRRFIP1_A23E_, LRRFIP1_A37E_), did not diminish binding to CT226 (Fig. S6B), ruling out these models.

To test the remaining two models, we focused on predicted salt bridges between LRRFIP1 residues R31 and R34 and CT226 D127 (Fig. 3C and D), as the distance between R31 or R34 and D127 are predicted to be close enough to form a salt bridge. Salt bridge interactions can be experimentally validated through mutational analysis. Changing the charge on one residue in the salt bridge is predicted to disrupt the interaction, while introducing a compensatory “charge flip” mutation of the second residue in the salt bridge is predicted to re-establish the salt bridge and should, therefore, at least partially restore function. Substitution of LRRFIP1 basic residues R31 and R34 with an acidic residue (LRRFIP1_R31E_ and LRRFIP1_R34D_) or substitution of CT226 acidic residue D127 with a basic residue (CT226_D127K_) disrupted binding to the cognate WT partner (Fig. 3E; Fig. S5B, lanes 2, 3, and 4 compared to lane 1). Importantly, co-expression of the double charge reversal mutants (LRRFIP1_R31E_-CT226_D127K_ or LRRFIP1_R34D_-CT226_D127K_) partially restored binding relative to LRRFIP1_WT_-CT226_D127K_ (Fig. 3E, lanes 5 and 6 compared to lane 4; Fig. S5C, lanes 2 and 3 compared to lane 1), with R31E showing a trend toward rescue and R34D reaching statistical significance. The observation that both LRRFIP1_R31E_-CT226_D127K_ and LRRFIP1_R34D_-CT226_D127K_ demonstrated even partial rescue leaves only one model consistent with the mutational results (Fig. 3D), as in the other model (Fig. 3C), the C-alpha distance between these two residues is greater than 10 Å, making a lysine–aspartate salt bridge unlikely (23). Using C-alpha distance cutoffs instead of terminal atom distances is more robust for *in silico* predicted structural models, as the long side chains are likely to be dynamic at fast time scales. For example, although R34 guanidine nitrogen is about 5.5 Å from oxygen on the D127 carboxyl group in our final model, a rotamer flip of the R34 would yield 3Å between the two. R34 is likely switching between the two rotamers on a fast time scale during the interaction but C-alpha distances throughout these dynamics would stay the same. We note that we likely do not see the full restoration of the interaction by a charge flip due to inherent asymmetry in the salt bridges and differences in length of the substituted residues. The fact that there is any restoration of binding over the single point mutants supports the close proximity of the two residues between the two proteins.

**Fig 5:**
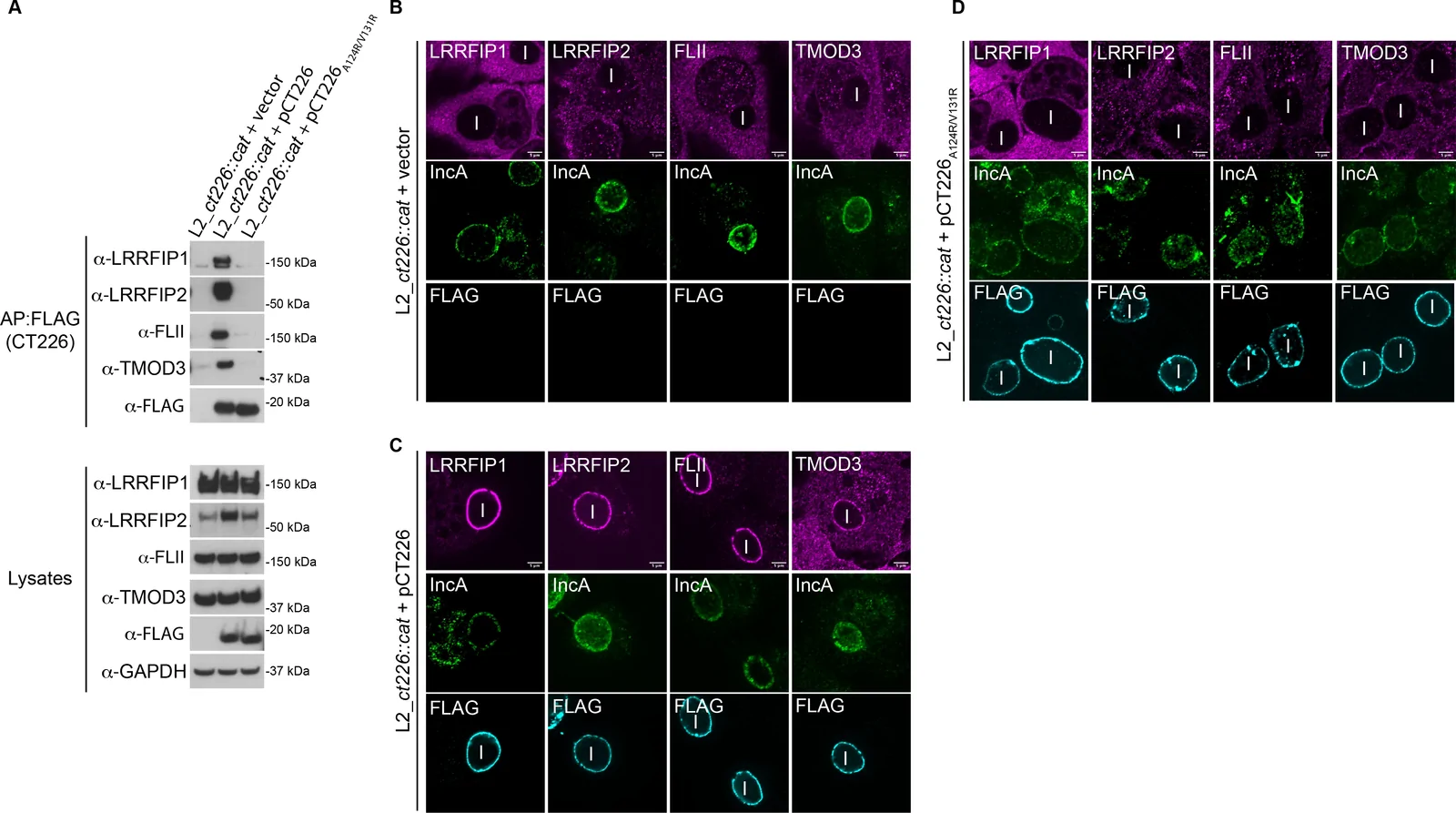
CT226 residues A124 and V131 are necessary for binding to the LFT complex and for LFT complex recruitment to the inclusion membrane. (**A through D**) HeLa cells were infected with either L2_*ct226::cat* + vector, L2_*ct226::cat* + pCT226, or L2_*ct226::cat* + pCT226_A124R/V131R_ for 24 h. Protein expression was induced at 1 hpi with aTc. (**A**) FLAG-tagged CT226 variants were affinity purified, followed by immunoblotting with the indicated antibodies to endogenous LFT proteins or to FLAG (CT226_FLAG_ variants). GAPDH serves as a loading control for lysates. Molecular weight markers (kDa) are indicated on the right. The LFT complex copurified with CT226 but not with the CT226_A124R/V131R_ variant. (**B through D**) Cells were fixed and stained with antibodies to endogenous LFT proteins (magenta) or IncA (green) to delineate the inclusion membrane, and FLAG (cyan, CT226_FLAG_ variants) to demonstrate inclusion membrane localization of CT226_FLAG_ variants. Samples were imaged by spinning disk confocal microscopy. Shown are single Z-slices. Scale bar = 5 µm. The LFT complex was recruited to the inclusion membrane in cells infected with the CT226 insertion mutant complemented with CT226 (**C**), but not with the control vector (**B**), or the CT226_A124R/V131R_ mutant, which cannot bind the LFT complex (**D**).

Altogether, these results are fully consistent with the final AlphaFold model (Fig. S7), increasing confidence that this structural prediction accurately represents the CT226_116-165_ and LRRFIP1_1-100_ complex structure. Notably, CT226 residues A124, V131, and D127 are conserved across all *Chlamydia* strains encoding CT226 (Fig. S1A). Similarly, LRRFIP1 residues R31 and R34 are conserved across mammalian species (Fig. S1B), including human, mouse, and pig, which are susceptible to the host-adapted *Chlamydia* species that encode CT226 homologs. This cross-species conservation underscores the functional importance of these residues and suggests evolutionary pressure to maintain this interaction interface.

### R31 and R34 are critical for LRRFIP1 binding to CT226 and for its recruitment to the inclusion during infection

After identifying LRRFIP1 R31 and R34 as key residues predicted to form a salt bridge with CT226, we next investigated whether these residues are required for binding CT226 during infection. HeLa cells were transfected with LRRFIP1_WT_-Strep, LRRFIP1_R31E_-Strep or LRRFIP1_R34E-_Strep, followed by infection with L2 expressing CT226_WT_-FLAG. Co-affinity purification revealed that mutation of either R31 or R34 abolished binding to CT226, despite higher expression levels of the mutant proteins compared to wild-type LRRFIP1 (Fig. 4A).

We next assessed whether these mutations affected LRRFIP1 recruitment to the inclusion. HeLa cells were transfected with FLAG-tagged LRRFIP1 variants, infected with L2, and inclusion localization was examined by immunofluorescence microscopy. Robust recruitment of LRRFIP1_WT_-FLAG (cyan) to the inclusion membrane was observed, whereas LRRFIP1_R31E_-FLAG or LRRFIP1_R34_D-FLAG failed to localize to the inclusion (Fig. 4B). Importantly, expression of the mutant LRRFIP1 proteins does not disrupt recruitment of endogenous LRRFIP1 (Fig. 4B, magenta), indicating that the mutants neither oligomerize with nor exert dominant-negative effects on the wild-type protein.

Together, these findings validate the structural model and demonstrate that LRRFIP1 residues R31 and R34 are critical for a stable interaction with CT226 during infection and for recruitment of LRRFIP1 to the inclusion during infection.

### CT226_A124R/V131R_ fails to bind the LFT complex during *C. trachomatis* infection and to recruit this complex to the inclusion

Having established that CT226 residues A124 and V131 are required for interaction with the LFT complex in transfection experiments, we tested whether they were required for LFT binding and recruitment during *C. trachomatis* infection. We generated a double mutant in which A124 and V131 were substituted with arginine (pCT226_A124R/V131R_) and introduced this mutant into the CT226 insertion mutant background (L2_*ct226::cat* + pCT226_A124R/V131R_).

We first assessed LFT binding by co-affinity purification in HeLa cells infected with L2_*ct226::cat* + vector, L2_*ct226::cat* + pCT226, or L2_*ct226::cat* + pCT226_A124R/V131R_. In contrast to CT226 mutant complemented with wild-type CT226, CT226_A124/V131R_ failed to co-purify with the LFT proteins (Fig. 5A). We next examined LFT recruitment to the inclusion by immunofluorescence microscopy (Fig. 5B through D). FLAG staining confirmed that both FLAG-CT226 and FLAG-CT226_A124/V131R_ localize to the inclusion. However, LFT protein recruitment was undetectable in cells infected with L2 expressing CT226_A124/V131R_, similar to the CT226 insertion mutant (L2_*ct226::cat* + vector). Together, these findings validate the structural predictions and demonstrate that CT226 residues A124 and V131 are essential for stable association of CT226 with the LFT complex and for the recruitment of the LFT proteins to the inclusion.

### CT226 and its interaction with LRRFIP1 influence late-stage inclusion size and infectious progeny recovery

To determine whether CT226 contributes to *C. trachomatis* development, we first quantified monolayer-associated infectious progeny production (see Materials and Methods) in HeLa cells at 48 and 72 hpi. In this initial assay, the CT226 insertion mutant unexpectedly exhibited increased recovery of infectious progeny from infected monolayers compared to L2 (Fig. S8A). This increase was modest at 48 hpi (~1.5 fold) but became more pronounced and statistically significant at 72 hpi (~2 fold increase at 72 hpi; *P* = 0.0472), suggesting that loss of CT226 is associated with increased infectious progeny recovery at later stages of infection.

**Fig 6:**
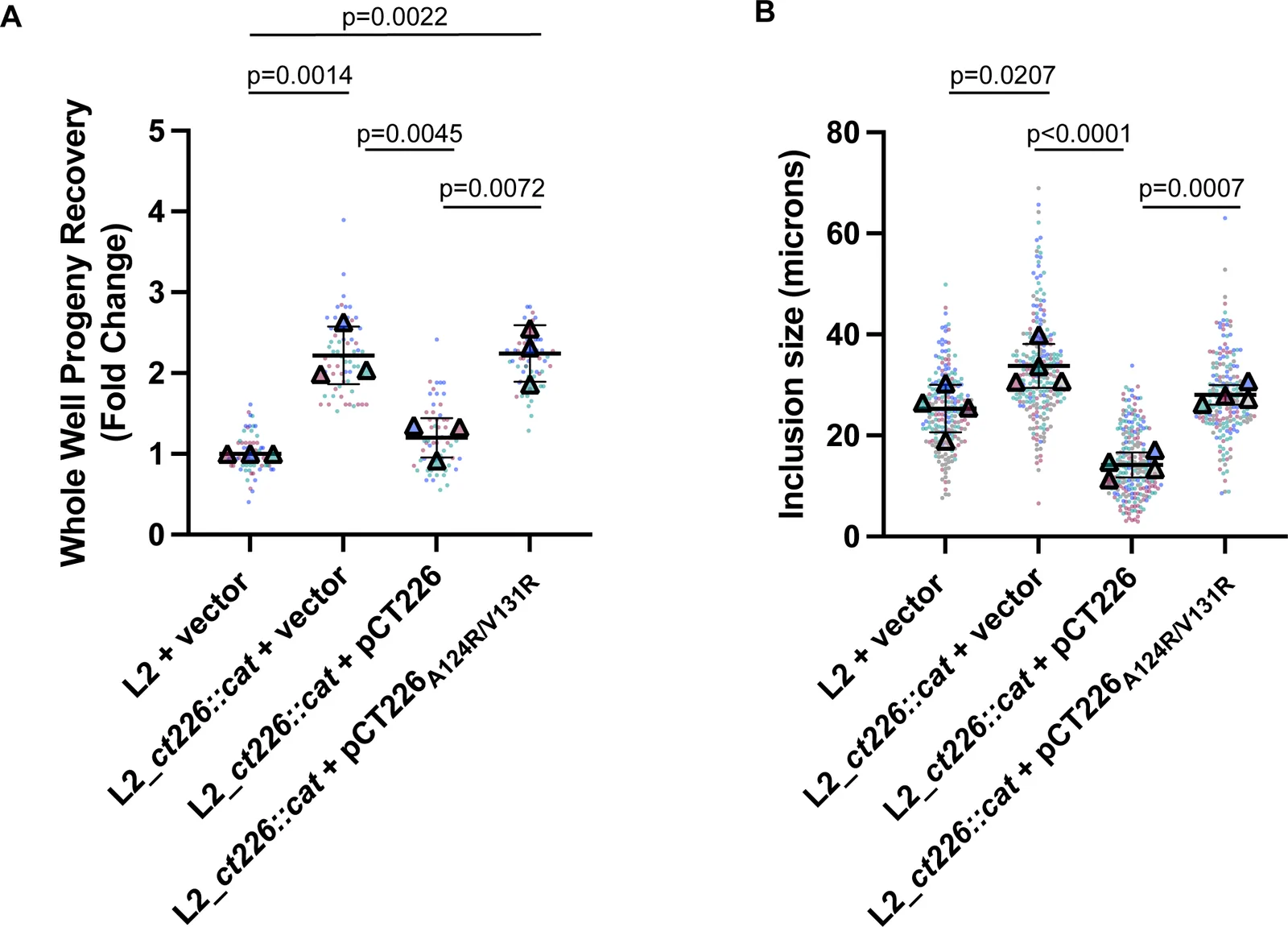
Loss of CT226 alters inclusion size and infectious progeny recovery. (**A and B**) HeLa cells were infected with L2 + vector, L2_*ct226::cat* + vector, L2_*ct226::cat* + pCT226, or L2_*ct226::cat*+pCT226_A124R/V131R_ for 72 h. Expression of CT226 variants was induced with aTc at 1 hpi. Whole-well infectious progeny recovery (**A**) and inclusion size (**B**) were quantified as described in Materials and Methods. (**A**) Whole well-infectious progeny recovery was normalized to L2 + vector and shown as fold change from three independent experiments. Each small dot represents an individual field color-coded by experiment. Large triangles represent the calculated mean value for each independent experiment. The black horizontal lines and error bars indicate the mean ± SD of the independent experiments. Statistical differences between groups were evaluated using a nested one-way ANOVA, with fields nested within each biological replicate, followed by Tukey’s multiple comparison test. *P* values are indicated. (**B**) Inclusion size across four independent experiments. Each small dot represents an individual inclusion color-coded by experiment. Large triangles represent the calculated mean value for each independent experiment. For each experiment, >30 inclusions were quantified per condition. The black horizontal lines and error bars indicate the mean ± SD of the independent experiments. Statistical differences between groups were evaluated using a nested one-way ANOVA, with inclusions nested within each biological replicate, followed by Tukey’s multiple comparison test. *P* values are indicated.

Given that the progeny phenotype was more pronounced at 72 hpi, we further analyzed the CT226 insertion mutant complemented with wild-type CT226 or CT226 variants at this later time point. Because progeny measurements at late time points can be influenced by bacterial release into the culture medium, we performed whole-well progeny assays in which both the infected monolayer and culture supernatant were collected and pooled prior to titration (see Materials and Methods). The CT226 insertion mutant harboring the empty vector displayed an ~2 fold increase in recovered infectious progeny compared to L2 + vector (Fig. 6A; *P* = 0.0014). Complementation with wild-type CT226 reduced infectious progeny recovery compared to the CT226 insertion mutant. These data indicate that CT226 limits recovery of infectious progeny at late time points.

We next examined whether the increased recovery of infectious progeny was accompanied by changes in inclusion size and whether this effect was dependent on CT226. At 72 hpi, the CT226 insertion mutant exhibited a modest but significant increase in inclusion size relative to L2 + vector (Fig. 6B; *P* = 0.0207). Representative immunofluorescence images showing inclusion morphology and host cell margins are included in Fig. S8B. Complementation with wild-type CT226 reduced inclusion size compared to the CT226 insertion mutant (L2_*ct226::cat* + vector, *P* < 0.0001), indicating that loss of CT226 is associated with increased inclusion size.

Finally, we tested whether the phenotypes associated with loss of CT226 required the CT226:LRRFIP1 interaction. In contrast to complementation with wild-type CT226, complementation with the LRRFIP1-binding-deficient mutant (CT226_A124R/V131R_) failed to reduce infectious progeny recovery (Fig. 6A) or inclusion size (Fig. 6B). Importantly, wild-type CT226 and CT226_A124R/V131R_ were expressed at similar levels (Fig. 5A, anti-FLAG immunoblot of lysates). This key control establishes that the differential ability of wild-type CT226 compared to the LRRFIP1 binding-deficient CT226 mutant to recruit the LFT complex is not attributable to differences in protein abundance but instead supports a direct role for the CT226:LRRFIP1 interaction in modulating inclusion size and recovery of infectious progeny. Together, these data support that CT226 and its interaction with LRRFIP1 limits inclusion size and infectious progeny recovery at late stages of infection.

## DISCUSSION

The *Chlamydia* developmental cycle requires precise reprogramming of host processes, including cytoskeletal remodeling, vesicular trafficking, and immune signaling, to survive in the hostile intracellular environment. Incs are central to this process, as they project from the inclusion membrane into the host cytoplasm and engage specific host proteins or multiprotein complexes. We previously reported that transfected CT226 co-affinity purifies the host LFT complex (12), and multiple independent *C. trachomatis* inclusion proximity-based and proteomic screens subsequently identified the LFT proteins at the inclusion (24–26). While our work was in progress, it was reported that CT226 co-affinity purifies with LRRFIP1 and that LRRFIP1 recruitment is lost upon CT226 knockdown (27), which is consistent with our identification of LRRFIP1 as a CT226-interacting protein. Here, we advance our understanding of the CT226:LFT complex interaction by defining the molecular basis of CT226-mediated LFT recruitment and assessing its functional consequences during infection. Our work reveals a role for the CT226:LRRFIP1 interaction in modulating the intracellular developmental cycle at late time points.

Our data demonstrate that CT226 directly binds LRRFIP1 through a compact, electrostatic interface involving the CT226 CC domain and the LRRFIP1 N-terminal helix. We used AlphaFold Multimer (21, 22) to generate an ensemble of atomic structural models for the CT226:LRRFIP1 complex. Through extensive experimental mutational validation, we were able to narrow in on a single model. This model suggests an electrostatic interaction within the LRRFIP1 CC interface that is stabilized by residue D127 in CT226 and residues R31 and R34 in LRRFIP1. Mutation of these residues abolished binding, while reciprocal charge-reversal mutations partially restored the interaction, validating the *in-silico* model. Importantly, these same residues in LRRFIP1 were required for CT226-mediated LFT recruitment to the inclusion during infection. Similarly, substitution of CT226 residues A124 and V131 with bulky amino acids disrupted both binding and inclusion localization of the LFT complex. Notably, these interface residues are conserved among CT226-encoding *Chlamydia* species and across their respective mammalian LRRFIP1 orthologs, consistent with co-evolution between the host-adapted *Chlamydia* species and their cognate hosts.

Although protein solubility limitations prevented assessment of direct binding between CT226 and LRRFIP2 or FLII, the strong homology between LRRFIP1 and LRRFIP2 and their shared interaction with FLII (15, 17) support a hierarchical assembly model in which CT226 engages LRRFIP1/2, which in turn recruits FLII to the inclusion. Since TMOD3 does not bind CT226 directly, it likely associates with the inclusion through interactions with LRRFIP1/2 and FLII (28, 29). We postulate that CT226 functions as an inclusion-localized scaffold that nucleates the LFT complex assembly.

A recent study reported that FLII recruitment to the inclusion was not diminished upon LRRFIP1 knockdown (27), leading to the suggestion that FLII recruitment may occur independently of LRRFIP1. However, because LRRFIP2 depletion was not assessed in that study, functional redundancy between LRRFIP paralogs could explain the persistence of FLII recruitment. Given the conserved CC-mediated interactions between LRRFIP1/2 and FLII (15, 17), it seems likely that LRRFIP2 recruits FLII when LRRFIP1 is depleted, explaining the observation that FLII still localizes at the inclusion.

Unexpectedly, our work reveals that loss of CT226 is associated with modest but statistically significant increases in inclusion size and recovery of infectious progeny, with the strongest differences observed at 72 hpi. Although a previously published study did not detect a change in *C. trachomatis* intracellular growth and development following CT226 depletion at 48 hpi (27), our data suggest that CT226-dependent effects on inclusion morphology and infectious progeny recovery may become more apparent at later stages of infection.

Complementation of the CT226 insertion mutant with wild-type CT226 reduced inclusion size and infectious progeny recovery, whereas complementation with the LRRFIP1 binding-deficient mutant did not produce the same effect. Reduced inclusion size following ectopic expression of CT226 has been reported previously (27, 30). In one study, the underlying mechanism was not defined (27), while the other study attributed the phenotype to metabolic consequences of Inc overexpression (30). Although the L2_*ct226::cat* strain was generated by insertional mutagenesis and, therefore, may be subject to polar effects on neighboring genes, we interpret phenotypes associated with this strain in the context of complementation and structure-function analyses. Because the LRRFIP1-binding-deficient mutant was expressed at levels comparable to wild-type CT226 but failed to reduce inclusion size or infectious progeny recovery, our data support a specific contribution of the CT226:LRRFIP1 interaction to these late-stage phenotypes.

Several models could account for the effect of the CT226:LFT interaction at late time points during infection. In one scenario, CT226-mediated recruitment of the LFT complex to the inclusion could transport the LFT complex to a new location where its activity influences inclusion morphology and progeny recovery. In contrast, LFT has little impact on these phenotypes in its native subcellular location. Alternatively, relocalization of the LFT complex to the inclusion from its normal subcellular location could sequester LFT proteins away from native host functions that affect infection outcome. Consistent with a modulatory role for LFT in this context, depletion of LRRFIP1 or the TMOD3 regulator, b-tropomyosin (16), has been reported to enhance infectious progeny production (25), supporting the model that LFT activity can alter late-stage phenotypes under certain conditions. Dissecting the downstream host pathways involved will require future targeted studies.

What advantages might *C. trachomatis* gain by encoding an Inc that modulates late-stage infection phenotypes? One possibility is that CT226-mediated recruitment of the LFT complex helps coordinate inclusion expansion, bacterial development, host cell viability, immune signaling, and/or metabolic capacity. By influencing these processes, CT226 could help tune the intracellular developmental cycle to the availability of host resources needed for bacterial replication and inclusion membrane expansion while limiting excessive host cell stress. Such coordination may be particularly important for obligate intracellular pathogens, which must balance replication with maintenance of the host cell environment. Consistent with this idea, *Coxiella burnetii* (31, 32) and *Rickettsia* species (33) coordinate intracellular replication with host cell integrity rather than simply maximizing growth. In addition, *Legionella pneumophila* deploys effectors that inhibit host translation, thereby activating stress signaling pathways that reshape the intracellular environment and influence bacterial replication (34, 35). Thus, CT226-mediated recruitment of the LFT complex may represent one mechanism by which *C. trachomatis* modulates host cell processes during late stages of infection.

The LFT proteins have been implicated in regulating cytoskeletal remodeling, transcriptional control, and innate immune signaling (14–16), processes that could either support or constrain *C. trachomatis* infection. LRRFIP1/2 link actin dynamics to transcriptional and innate immune pathways; FLII modulates actin and membrane-associated processes; and TMOD3 regulates actin filament stability. CT226-mediated recruitment of LFT to the inclusion may alter local cytoskeletal organization or stress-responsive signaling in ways that limit inclusion expansion, developmental timing, or production of infectious progeny. In this way, CT226 may spatially and temporally redirect LFT-associated activities at the inclusion membrane, providing a potential mechanism by which *C. trachomatis* coordinates the intracellular developmental cycle with host cell processes.

While our studies have focused on the role of the LFT complex in regulating *C. trachomatis* intracellular growth in cultured cells, the LFT complex may additionally influence tissue pathology during *C. trachomatis* infection. *In vivo*, infection induces epithelial injury and chronic inflammation that resolves through fibrosis rather than regenerative repair, producing tissue damage that results in infertility and blindness (36, 37). These pathologies are driven by sustained cytokine signaling, prolonged immune cell recruitment, excessive deposition of extracellular matrix by fibroblasts, and bacterial subversion of TLR, NF-kB, and inflammasome-associated pathways that maintain a non-healing wound-like state (3, 38–40). Because LFT proteins regulate cytoskeletal dynamics, innate immune signaling, and wound healing pathways implicated in these processes (14–16), CT226-mediated sequestration of LFT may contribute to impaired tissue repair and persistent inflammation *in vivo*, thereby favoring fibrosis (36, 37). Determining whether CT226 influences tissue remodeling *in vivo* will require dedicated animal studies beyond the scope of the present work.

Future work should define how CT226 binding to the LFT complex modulates host trafficking, signaling, immune evasion, and tissue-level pathogenesis. One intriguing possibility is that CT226 mimics the binding surface of a native LRRFIP1/2 binding partner, thereby disrupting a native host interaction. Identifying such native interactions would not only clarify the biological significance of the CT226:LFT interaction during infection but could also deepen our understanding of LFT functions and how their dysfunction contributes to disease. Alternatively, CT226 engagement of LFT functions may enhance or redirect their interactions in ways that generate novel functions at the inclusion, expanding the capacity of *C. trachomatis* to modulate host responses.

In summary, our findings identify CT226 as an inclusion-localized scaffolding hub that redirects the host multiprotein LFT complex to the inclusion membrane through a compact, electrostatic interaction with LRRFIP1, and potentially LRRFIP2. By defining the molecular interface required for CT226-mediated LFT recruitment, this work provides a framework for dissecting how Incs engage host multiprotein complexes at inclusion membrane. Our data further link the CT226:LRRFIP1 interaction to limiting inclusion size and production of infectious progeny at late time points of infection, representing the first example of a *C. trachomatis* Inc shown to constrain the intracellular developmental cycle.

### Study limitations

First, as is common in mechanistic studies of *Chlamydia*, experiments were performed in a transformed epithelial cell line, which does not encompass the broader immune and tissue context of *in vivo* infection. Second, direct binding between CT226 and LRRFIP2 or FLII could not be assessed due to protein solubility constraints, and additional biochemical approaches may be required to fully define the assembly hierarchy. Third, while mutational and charge-reversal analyses strongly support the predicted CT226:LRRFIP1 interface, it is still just a model, and high-resolution structural studies will ultimately be required to define the CT226:LRRFIP1 architecture.

## MATERIALS AND METHODS

### Mammalian cell culture and bacterial propagation

HeLa and Vero cells were obtained from American Type Culture Collection (ATCC). HeLa cells were cultured and maintained in Eagle’s minimum essential medium (MEM; University of California, San Francisco [UCSF] Cell Culture Facility) supplemented with 10% (vol/vol) fetal bovine serum (FBS) from Gemini at 37°C in 5% CO_2_. HEK293T cells (gift from Nevan Krogan, University of California, San Francisco) and Vero cells were cultured and maintained in Dulbecco’s modified Eagle’s medium (DMEMl UCSF Cell Culture Facility) supplemented with 10% (vol/vol) FBS at 37°C in 5% CO_2_. Cells were routinely tested for mycoplasma (Sigma).

*C. trachomatis* L2 and derivative strains were propagated in Vero cells and harvested at 48 h post infection (hpi) by osmotic lysis. Bacteria were pelleted by centrifugation at 14,000 × *g*, resuspended in SPG (75g/L sucrose, 0.5 g/L KH_4_HPO_4_, 1.2 g/L Na_2_HPO_4_, 0.72 g/L glutamic acid, pH 7.5), and stored as single use aliquots at −80°C.

HeLa cells were used for infection experiments, whereas HEK293T cells were used for ectopic expression studies.

Stellar chemically competent *Escherichia coli* (Takara Bio) was used for plasmid propagation for mammalian expression. 10-Beta *E. coli* (NEB) and dam^−^/dcm^−^ chemically competent *E. coli* (NEB) were used to produce unmethylated plasmids for transformation into *C. trachomatis. E. coli* BL21 (DE3) was used for recombinant protein expression.

### Antibodies and reagents

Primary antibodies were obtained from the following sources: rabbit anti-LRRFIP1 (Abcam, ab229466), mouse anti-LRRFIP1 (Santa Cruz Biotechnology, sc-514221), rabbit anti-LRRFIP2 (Atlas, HPA042023), rabbit anti-FLII (Invitrogen, PA5-82280), rabbit anti-TMOD3 (Invitrogen, PA5-51645), mouse anti-FLAG (Sigma, F3165), rabbit anti-FLAG (Sigma, F7425), goat anti-FLAG (Novus Biologicals, NB600-344), mouse anti-GAPDH (Millipore, MAB374), goat anti-MOMP L2 (Fitzgerald, 20C-CR2104GP), and rabbit anti-Strep TagII HRP (Millipore, 71591-3). Mouse anti-IncA was kindly provided by Dan Rockey (Oregon State University). Goat anti-IncA was kindly provided by Fabienne Paumet (Thomas Jefferson University). Rabbit anti-IncE was previously described (41).

Alexa Fluor-conjugated donkey secondary antibodies (Life Technologies) were used for immunofluorescence microscopy.

Primary antibodies were used at the following dilutions for immunoblotting and immunofluorescence, respectively: anti-LRRFIP1 (1:1,000 and 1:300), anti-LRRFIP2 (1:1,000 and 1:100), anti-FLII (1:1,000 and 1:100), anti-TMOD3 (1:1,000 and 1:100), anti-Strep TagII HRP (1:1, 1,000), all anti-FLAG (1:3,000 and 1:200), anti-GAPDH (1:20,000), anti-MOMP (1:5,000 and 1:500), anti-anti-IncA (1:500 and 1:100), IncE (1:500 and 1:100), and CT226 (1:100 and 1:1,000).

Primers were synthesized by Integrated DNA Technologies or by Elim Biopharm. They are available upon request.

### Insertional mutagenesis of CTL0478

The homolog of CT226 from *C. trachomatis* Serovar D is CTL0478 in L2 (434/Bu). The gene *ctl0478* in L2 was disrupted using TargeTron technology (19). The CTL0478 gene sequence was scanned for potential target insertion sites using the TargeTron algorithm (Sigma-Aldrich). The predicted target site closest to the start codon of CTL0478 (between nucleotides 415 and 416) was chosen. The primers IBS-CTL0478 (5′-AAAAAAGCTTATAATTATCCTTACTAAACCTTTGCGTGCGCCCAGATAGGGTG-3′), EBS1d-CTL0478 (5′-CAGATTGTACAAATGTGGTGATAACAGATAAGTCCTTTGCTCTAACTTACCTTTCTTTGT-3′), EBS2-CTL0478 (5′-TGAACGCAAGTTTCTAATTTCGGTTTTTAGTCGATAGAGGAAAGTGTCT-3′), and EBS universal (Sigma-Aldrich) were used to retarget vector pDFTT3-CAT (42) for disruption of CTL0478. The resulting vector pDFTT3-CAT-CTL0478 was transformed into *C. trachomatis* L2/434/Bu using the CaCl_2_-mediated approach (18), with modifications described in reference 43. Transformed bacteria were selected in the presence of 0.5 µg/mL chloramphenicol (Sigma-Aldrich) beginning at 12 hpi and plaque-purified in the presence of 1 µg/mL chloramphenicol. Intron insertion at the correct target sites was verified by PCR (Fig. S2B) using primers CTL0478_F/R (5′-TTTGCTGAACAACAATTCCC-3′ and 5′-TACTACAAGCCGTAAAGAGC-3′) and by sequencing of the resulting PCR product (Eton Bioscience). Loss of CTL0478/CT226 protein was verified by western blot analysis (Fig. S2C).

### Plasmid construction

#### Plasmids used in co-transfection and infection experiments

The CTL0478 constructs used for ectopic expression studies were PCR amplified from L2 genomic DNA and cloned into the EcoRI and NotI sites in pcDNA4.0/2×StrepII. Point mutants were generated by overlapping PCR or site-directed mutagenesis. A CTL0478 A124R and V131R double mutant was generated using a G-block fragment (Integrated DNA Technology) as the template. The resulting plasmids express CTL0478 variants with a 2×StrepII epitope fused to the C-terminus.

Human LRRFIP1 and TMOD3 were kind gifts from Dr. Sourav Bandyopadhyay (University of California, San Francisco) (44) and shuttled by Gateway cloning (Thermo Fisher Scientific) to destination vectors encoding either a C-terminal 3×FLAG or a 2×StrepII tag (kind gifts of Dr. Nevan Krogan, University of California, San Francisco). All LRRFIP1 point mutants were generated by site-directed mutagenesis (QuickChange, Agilent).

#### Plasmids used to generate L2 strains

##### pBOMB4-based plasmids

The CTL0478 gene was PCR amplified from L2 genomic DNA and cloned into the NotI and SalI sites in the pBOMB4 shuttle vector (kindly provided by Ted Hackstadt, Rocky Mountain Laboratory, and Mary Weber, University of Iowa Carver College of Medicine) (45, 46).

##### p2TK2-SW2-based plasmids

The *E. coli/Chlamydia* shuttle vector p2TK2-SW2-mCherry (a kind gift of Dr. Isabelle Derre, University of Virginia) (47) was modified by removing the *mCherry* gene by InFusion (Takara Biotech) cloning to generate p2TK2-SW2. For plasmids that were transformed into *C. trachomatis* L2 strains, CTL0478 variants were PCR amplified from the corresponding CTL0478-encoding plasmid (described above for co-transfection experiments) and cloned into EagI and SbfI sites in the p2TK2-SW2 plasmid.

##### Plasmids used for expression and purification of proteins in *E. coli*

LRRFIP1, LRRFIP2, FLII, TMOD3, and CTL0478 ORFs and deletion constructs were PCR amplified from constructs described above and inserted into pH3C-LIC and pHM3C-LIC using ligation-independent cloning to generate proteins with N-terminal 6×HisMBP or 8×His fusions. Construction of the plasmid encoding 6×His-MBP-IncE_101-132_ was previously described (12).

All plasmids were sequence verified.

### Generation of L2 strains

Plasmids were isolated from dam^−^/dcm*^−^ E. coli* and transformed into L2 or L2_ct226::cat as previously described with slight modifications (47). Briefly, 10 µg of plasmid DNA was incubated with 1 × 10^7^ infection-forming units (IFUs) of L2 in 200 µL transformation buffer (10 mM Tris [pH 7.4] in 50 mM CaCl_2_) for 30 min incubation at room temperature. The transformation mix was then added to 12 mL of MEM supplemented with 10% FBS. Two milliliters of suspended bacteria was added to each well of a six-well plate containing Vero cells (2 × 10^5^/well). Plates were centrifuged at 1,000 RPM for 30 min at 4°C, followed by incubation at 37°C in 5% CO_2_ for 30 min. The infection media were replaced with fresh media containing 10 µg/mL ampicillin (Sigma) and cycloheximide (1 µg/mL, Sigma) to select for transformed L2. After 3 passages, clonal transformants were isolated by plaque purification.

L2 overexpressing IncG_FLAG_ was a generous gift from Dr. Isabelle Derre (University of Virginia).

### Affinity purifications

For transfection studies with HEK293T cells, 6 × 10^6^ cells were seeded in each of two to three 10 cm^2^ plates and transfected with the indicated plasmids using Continuum (GeminiBio) or FuGENE (Promega) transfection reagent following the manufacturer’s instructions. At 48 h post-transfection, cells were scraped in PBS, pelleted, lysed in 1 mL of ice-cold lysis buffer (50 mM Tris [pH 7.4], 150 mM NaCl, 1 mM EDTA, 0.5% Igepal (Sigma), Roche Complete protease, and PhosSTOP phosphatase inhibitor) at 4°C for 20 min while rotating, and centrifuged at 5,700 RPM at 4°C for 20 min. Lysates were incubated overnight with 60 µL of Strep-Tactin Sepharose beads (IBA) or anti-FLAG M2 magnetic beads (Sigma) on a nutator. Beads were washed three times in 1 mL of lysis buffer containing 0.05% Igepal. Samples were eluted in 45 µL of 2.5 mM D-desthiobiotin (IBA) or 150 µg/mL FLAG peptide (Sigma) in lysis buffer for 30 min on a rocker at room temperature. Eluates were analyzed by immunoblotting with the indicated antibodies.

For infection studies in HeLa cells, 3 × 10^5^ cells were seeded in each well of two six-well plates. Cells were left untransfected or transfected with the indicated plasmid using Continuum (GeminiBio) or FuGENE (Promega) transfection reagent following the manufacturer’s instructions. At 24 h post-transfection, HeLa cells were infected with the indicated L2 strains by centrifugation at 1,000 RPM for 30 minutes at 4°C followed by incubation at 37°C in 5% CO_2_ for 30 min. Infection media were removed, and fresh media containing 50 ng/mL anhydrous tetracycline (aTC; Takara biotech) were added to infected cells to induce expression of IncG _FLAG_ or CT226_FLAG_ variants. At 24 h post-infection (hpi), cells were subjected to affinity purification as described above. Eluates were analyzed by immunoblotting with the indicated antibodies.

### *In vitro* pulldowns

His-tagged proteins expressed in *E. coli* BL21 (DE3) were purified over Ni-NTA (Gold Biotechnology). *In vitro* pulldown experiments were performed as follows: purified 6×His-MBP-CT226_116-165_-Strep, 8×His-CT226_116-165_-Strep, or 6×His-MBP-IncE_101-132_-Strep proteins were immobilized on Strep-Tactin Sepharose beads (IBA), subsequently incubated with purified 8×His-LRRFIP1 or 8×His-TMOD3 constructs (1:2 molar ratio); the beads were washed extensively; the complexes were eluted by boiling, and then analyzed by Coomassie stain.

### Immunofluorescence microscopy

HeLa cells grown on 24-well glass bottom plates (MatTek) were infected with the indicated L2 strains as follows: L2 suspended in MEM supplemented with 10% FBS were centrifuged onto cell monolayers at 1,000 RPM for 30 min at 4°C, followed by incubation at 37°C in 5% CO_2_ for 30 min. Infection media were replaced with fresh media containing 50 ng/mL aTC to induce expression of CT226_FLAG_ variants, and cells were incubated at 37°C in 5% CO_2_. For experiments assessing localization of LRRFIP1_FLAG_ variants, cells were first transfected with the indicated LRRFIP1_FLAG_ variant using Effectene (Qiagen) following the manufacturer’s instructions, and at 24 h post-transfection, cells were infected with the indicated L2 strain. Experiments assessing inclusion size were performed at low MOI (MOI ~0.5–1) to eliminate the possibility that more than one inclusion would enter per cell and undergo fusion to ensure accurate measurements. Cells were fixed at 72 hpi and processed for IF with antibodies targeting IncA, FLAG, and MOMP. Inclusion size was quantified as described below. For experiments quantifying production of infectious progeny, EBs were harvested at 48 or 72 hpi from primary infections (as described below) performed at MOI ~5. The harvested EBs were then used to infect fresh HeLa cells plated on glass bottom plates at varying MOIs. At 24 hpi, the cells were fixed in 4% PFA in PBS for 15 min at room temperature, and then permeabilized in 0.2–0.5% Triton X-100 in PBS for 5 min at room temperature. Cells were blocked in PBS containing 2–5% BSA for 1 h, and then stained with the indicated primary and fluorophore-conjugated secondary antibodies in 2–5% BSA for 1 h each. Cells were mounted in Vectashield mounting media with or without DAPI (Vector Laboratories) and imaged on a laser scanning disc confocal microscope. Images were acquired using Yokogawa CSU-X1 spinning disk confocal mounted on a Nikon Eclipse Ti inverted microscope equipped with an Andor Clara digital camera and CFI APO TIRF 60× oil or PLAN APO 40× objective. Single Z slices were acquired for images used for protein localization and quantifying production of infectious progeny. Images were acquired by NIS-Elements Software 4.10 (Nikon). For each set of experiments, the exposure time for each filter set for all images was identical. Images were processed with Nikon Elements (Nikon) or FIJI (ImageJ, NIH) software.

### Quantitation of inclusion size

To quantify inclusion size, HeLa cells infected with the indicated L2 strains for 72 h were fixed with 4% PFA and stained with anti-IncA or anti-FLAG together with anti-MOMP antibodies, followed by staining with fluorescent secondary antibodies. Images were acquired under identical microscope settings within each experiment. Inclusion size was measured using FIJI (ImageJ, NIH). Linear dimensions were measured using the Straight Line tool on the IncA or FLAG channel to delineate the inclusion membrane. A line was manually drawn across the longest axis of each inclusion. Images were inspected at high magnification to ensure accurate placement of line endpoints at inclusion boundaries. Measurements were obtained using analyze → measure, and the length parameter (µm) was recorded. For each condition, at least 30 independent inclusions were measured per biological replicate. All images within a data set were analyzed using identical calibration and measurement settings. Inclusion measurements (µm) from four independent biological replicates are displayed as individual dots and color-coded, with biological replicate means indicated as triangles. Statistical comparisons between groups were performed as described in the Statistical Analysis section.

### Quantitation of infectious progeny production and recovery

For initial comparisons of infectious progeny production between parental L2 and L2_*ct226::cat*, infected HeLa cell monolayers were harvested at 48 and 72 hpi. The culture medium was removed, and monolayers were then osmotically lysed in ddH2O to release bacteria. For complementation and CT226 interface analyses, infectious progeny was quantified using whole-well sampling at 72 hpi. For these experiments, the culture medium was collected, and the cells were osmotically lysed in ddH_2_O. The culture medium was then mixed with the water-lysed monolayer, representing the whole-well sample that captures both released and cell-associated infectious bacteria. Samples were serially diluted and used to infect fresh HeLa monolayers for enumeration of infectious progeny. Following removal of the inoculum, fresh medium containing 1 mg/mL heparin (Sigma) was added to cells. At 24 hpi, cells were fixed in 4% PFA and stained with anti-MOMP antibodies, followed by fluorescent secondary antibodies. For each condition, 10 fields of view were acquired per coverslip with two technical replicates (10–20 total fields per condition). Inclusions were manually quantified based on MOMP staining. Progeny values were normalized to the matched control condition (either L2 or L2 + vector) within the same biological replicate and represented as fold -change relative to the respective control. Statistical comparisons between groups were performed as described in “Statistical analysis” below.

### Quantitation of CT226-LRRFIP1 variant interactions and charge reversal rescue

Densitometric analysis of western blots was performed using FIJI (ImageJ, NIH). A rectangular region of interest of equal size was drawn around each band of interest. The integrated density was measured for each band. For normalization, the intensity of each target protein band (i.e., LRRFIP1 variant) was divided by the intensity of the corresponding CT226 variant (i.e., CT226 D127K) band from the same lane. Ratios were then normalized to the indicated control condition (set to 1.0) to allow comparison across blots. Three independent replicates were quantified for each experiment. Statistical comparisons between groups were performed as described in “Statistical analysis” below.

### Structural prediction using AlphaFold

CT226 (residues 116–162)–LRRFIP1 (residues 1–100) structural models were generated with AlphaFold-Multimer (v3) run via ColabFold (version 1.5.2) using default parameters (five models, 20 recycles) with AMBER relaxation step. Predictions were also run using AlphaFold3 but discarded based on low PAE scores and manual examination of the interface. Models were examined, and figures were prepared using ChimeraX (48).

### Bioinformatics

The full-length CT226 protein sequence from L2 (strain 434/Bu; CTL0478), *C. trachomatis* serovar D (D/UW-3/Cx; CT_226), *C. trachomatis* serovar A (A/HAR-13; CTA_0248), *C. muridarum* (MoPn/Nigg; TC_0497), and *C. suis* (MD56; ESN89580.1) were analyzed with SMART (49) using standard parameters to predict the coiled-coiled domain in CT226. Multiple sequence alignment of CT226 homologs was performed using Clustal Omega (50).

### Statistical analysis

For each experiment, three or more independent biological replicates were performed, and the results are plotted individually or combined as described in the figure legends. For experiments with naturally high variability due to the asynchronous nature of L2 infections, Superplots were used to represent the data (Fig. 6 and Fig. S8). Graphs were generated using GraphPad Prism 10.6.1. Statistical analysis was performed using nested one-way analysis of variance (ANOVA), followed by Tukey’s multiple-comparison test, nested *t*-test, or paired *t*-test, as indicated in the figure legends.

## SUPPLEMENTAL MATERIAL

Fig. S1 mbio.01953-26-s0001.tif Multiple sequence alignment of the predicted CC domain of *Chlamydia* CT226 homologs and the N-terminal helix of mammalian LRRFIP1 homologs.

Fig. S2 mbio.01953-26-s0002.tiff Generation and validation of the L2_ct226::*cat* TargeTron mutant.

Fig. S3 mbio.01953-26-s0003.tif The CT226 CC domain interacts directly with LRRFIP1 but not with TMOD3.

Fig. S4 mbio.01953-26-s0004.tiff AlphaFold2 models of the CT226-LRRFIP1 interaction.

Fig. S5 mbio.01953-26-s0005.tiff Analysis of interactions between CT226 and LRRFIP1 point mutants.

Fig. S6 mbio.01953-26-s0006.tiff LRRFIP1 residues L15, A23, and A37 are dispensable for the CT226:LRRFIP1 interaction.

Fig. S7 mbio.01953-26-s0007.tiff Model of the CT226:LRRFIP1 interaction.

Fig. S8 mbio.01953-26-s0008.tiff Loss of CT226 results in increased progeny production and inclusion size.

Legends mbio.01953-26-s0009.docx Supplemental figure legends.

ASM does not own the copyrights to Supplemental Material that may be linked to, or accessed through, an article. The authors have granted ASM a non-exclusive, world-wide license to publish the Supplemental Material files. Please contact the corresponding author directly for reuse.
