# Supplementary material for: The Chlamydia trachomatis effector CT226 directly engages LRRFIP1 to recruit a multiprotein complex that limits inclusion size and progeny production
Source: mBio. 2026 Sep 17;17(10):e01953-26. doi: 10.1128/mbio.01953-26 (PMC13712786; doi:10.1128/mbio.01953-26)
Supplement: Supplementary file 1 [file mbio.01953-26-s0009.docx]

**SUPPORTING INFORMATION**

**S1 Fig. Multiple sequence alignment of the predicted CC domain of CT226 homologs from various *Chlamydia* strains and the N-terminal helix of LRRFIP1 homologs from various mammalian species.**

(A) Alignment of CT226 homologs. Residues A124, D127, and V131 (bolded in black) are conserved across all *Chlamydia* strains encoding CT226 and contribute to the CT226:LRRFIP1 interaction. The underlined region corresponds to the predicted CT226 CC domain across the *Chlamydia* strains. *C. trachomatis* is human-adapted; *C. muridarum* is mouse-adapted, and *C. suis* is pig-adapted. Strain names and locus tags of CT226 homologs are described in Materials and Methods. (B) Alignment of LRRFIP1 homologs. Residues mutated (R131 and R134) in this study are bolded in black. R31 and R34 are required for the CT226:LRRFIP1 interaction and are conserved across mammalian species that are susceptible to the *Chlamydia* species and serovars shown in panel A.

**S2 Fig. Generation and validation of L2_*ct226::cat* TargeTron mutant.**

(A) Schematic of CT226 domain structure. TM, transmembrane domain. CC, coiled coil. Red triangle, site of intron insertion. Arrows, PCR primer binding sites. (B) PCR verification of intron insertion at the targeted genomic site. Primers flanking the TargeTron insertion site (indicated by arrows, panel A) amplify the predicted 499 bp DNA fragment in L2 and the predicted 2388 bp fragment in L2_*ct226::cat*.

**S3 Fig. The CT226 CC domain interacts directly with LRRFIP1 but not with TMOD3**

(A) Purified Strep-tagged CT226_116-165_ fused to maltose binding protein (MBP) (orange asterisk) was immobilized to Strep-Tactin beads and incubated with purified His-tagged LRRFIP1 truncation variants (yellow asterisks). Complexes were resolved by SDS-PAGE and visualized by Coomassie blue stain. MBP-His-CT226_116-165_-Strep (orange asterisk) binds directly and specifically to His-LRRFIP1 (yellow asterisks) constructs containing the N-terminal helix. Molecular weight markers (kDa) are indicated on the left. Input and pulldown lanes are indicated. Pulldowns are also shown in Fig 2D. (B) Purified Strep-tagged CT226_116-165_ fused to His (orange asterisk) or purified Strep-tagged IncE_101-132_ fused to MBP-His (pink asterisk) was immobilized to Strep-Tactin beads and incubated with His-tagged LRRFIP1_1-249_ (yellow asterisk) or His-tagged TMOD3 (blue asterisk). Complexes were resolved by SDS-PAGE and visualized by Coomassie blue stain. His-CT226_116-165_-Strep (orange asterisk) binds directly and specifically to His-LRRFIP1_1-249_ (yellow asterisk). There was no enrichment for His-TMOD3 binding (blue asterisk) to immobilized His-CT226_116-165_-Strep (orange asterisk) compared to beads alone. No interaction was detected between MBP-His-IncE_101-132_-Strep (pink asterisk) and LRRFIP1_1-249_ (yellow asterisk). Molecular weight markers (kDa) are indicated on the left. Input and pulldown lanes are indicated. Pulldowns are also shown in Fig 2E.

**S4 Fig. AlphaFold2 models of CT226-LRRFIP1 interaction.**

(A) Two views of the five predicted models from AlphaFold2 as in Fig 3 (A) but not truncated to the helices, showing full predictions. (B) Same as in A but models colored by the pLDDT scores.

**S5 Fig. Analysis of interactions between CT226 and LRRFIP1 point mutants**

(A) Affinity purification of Strep-tagged CT226 single point mutants transiently expressed in HEK293T cells followed by immunoblotting with the indicated antibodies. GAPDH serves as a loading control for the lysates. Molecular weight markers (kDa) are indicated on the right. The CT226 mutants A124R and V131R fail to bind to the LFT proteins. (B) Binding of LRRFIP1 variants to CT226 variants was quantified by densitometric analysis of western blots (taken from Fig 3E, lanes 1-4). LRRFIP1 band intensities were normalized to the corresponding CT226 variant band in the same lane and expressed relative to the indicated control condition (LRRFIP1_WT_:CT226_WT_, set to 1.0) to calculate relative fold change in binding. Data represents the mean ± SD for three independent experiments. (C) Quantitation of charge reversal rescue experiment (taken from Fig 3E, lanes 4-6). Binding of LRRFIP1 variants to CT226_D127K_ was quantified by densitometric analysis of western blots. LRRFIP1 band intensities were normalized to the corresponding CT226_D127K_ band in the same lane and expressed relative to the indicated control condition (LRRFIP1_WT_:CT226_D127K_, set to 1.0) to calculate relative fold change in binding. Data represents the mean ± SD for three independent experiments. Statistical significance was assessed using paired *t*-test.

**S6 Fig. LRRFIP1 residues L15, A23, and A37 are dispensable for the CT226:LRRFIP1 interaction.**

(A) Three Alpha-Fold 2-generated models where mutations of residues L15, A23 and A37 on LRRFIP1, shown as yellow sticks, would lead to severe clashes. (B) Affinity purification of FLAG-tagged LRRFIP1 variants transiently co-expressed with Strep-tagged CT226, followed by immunoblotting with antibodies to FLAG (LRRFIP1 variants) and Strep (CT226). Molecular weight markers (kDa) are indicated on the right. LRRFIP1 variants L15E, A23E, or A37E did not disrupt binding to CT226, suggesting that these three models are unlikely.

**S7 Fig**. **Model of CT226:LRRFIP1 interaction.**

(A) Model which is consistent with the mutational analysis. CT226 ribbon is orange and LRRFIP1 ribbon is purple. Mutated residues are shown as sticks. Residues which interfere with binding when mutated are shown in yellow. Residues that retain binding when mutated are shown in green. (B) Zoomed in views of the mutated residues.

**S8 Fig. Loss of CT226 results in increased progeny production and inclusion size.**

(A) HeLa cells were infected with L2 or L2_*ct226::cat* for 48 or 72 hrs, and monolayer-associated infectious progeny was quantified as described in Methods. Data are normalized to L2 within each time point and shown as fold-change from three independent experiments. Each small dot represents an individual field, color-coded by experiment. Large triangles represent the calculated mean value for each independent experiment. The black horizontal lines and error bars indicate the mean +/- SD of the independent experiments. Statistical differences between groups at the indicated time points were evaluated using a Nested t-test, with fields nested within each biological replicate. P values are indicated.

(B) Representative fluorescent images of HeLa cells infected with the indicated strains for 72 hpi. Protein expression was induced at 1 hpi with aTc. Cells were fixed and stained with antibodies to IncA or FLAG (green) to delineate inclusions and demonstrate CT226 variant expression at the inclusion membrane, respectively, and co-stained with phalloidin (magenta) to delineate host cell margins. Samples were imaged by spinning disk confocal microscopy. Shown are single Z-slices. Scale bar= 5 µm.
